# Risk factors associated with structural postural changes in the spinal column of children and adolescents

**DOI:** 10.1016/j.rpped.2014.11.012

**Published:** 2015-03

**Authors:** Juliana Adami Sedrez, Maria Izabel Zaniratti da Rosa, Matias Noll, Fernanda da Silva Medeiros, Claudia Tarragô Candotti

**Affiliations:** Universidade Federal do Rio Grande do Sul (UFRGS), Porto Alegre, RS, Brazil

**Keywords:** Risk factors, Posture, Spine, Child, Adolescent, Epidemiology

## Abstract

**OBJECTIVE:**

To investigate the association between behavioral risk factors, specifically postural habits, with the presence of structural changes in the spinal column of children and adolescents.

**METHODS:**

59 students were evaluated through the self-reporting Back Pain and Body Posture Evaluation Instrument and spinal panoramic radiographic examination. Spine curvatures were classified based on Cobb angles, as normal or altered in the saggital plane and as normal or scoliotic in the frontal plane. Data were analyzed using SPSS 18.0, based on descriptive statistics and chi-square association test (a=0,05).

**RESULTS:**

The prevalence of postural changes was 79.7% (n=47), of which 47.5% (n=28) showed frontal plane changes and 61% (n=36) sagital plane changes. Significant association was found between the presence of thoracic kyphosis and female gender, practice of physical exercises only once or twice a week, sleep time greater than 10 hours, inadequate postures when sitting on a seat and sitting down to write, and how school supplies are carried. Lumbar lordosis was associated with the inadequate way of carrying the school backpack (asymmetric); and scoliosis was associated wuth the practice of competitive sports and sleep time greater than 10 hours.

**CONCLUSIONS:**

Lifestyle may be associated with postural changes. It is important to develop health policies in order to reduce the prevalence of postural changes, by decreasing the associated risk factors.

## Introduction

Static postural changes are considered a public health problem, especially those that affect the spinal column, as they may be a predisposing factor for degenerative conditions of the spine in adulthood;^1^^-^ 3 additionally, depending on their magnitude, they are capable of causing impairment to some daily activities.

The phases of childhood and adolescence correspond to those during which young individuals attend the school environment, where they remain for long periods in the seated position, usually assuming an inadequate posture, most often on inappropriate furniture,^4^ which in addition to the tendency to a sedentary lifestyle throughout school time^5^ can also favor the onset of static postural changes. Furthermore, there seems to be a trend that postural habits adopted in childhood and adolescence will continue into adulthood.^6^

Thus, investigations on the occurrence of static postural changes and the variables associated with this condition help to understand the risk factors for spinal problems, early detection of these changes being the first step towards prevention of conditions that predispose to the emergence of these disorders. Thus, early detection of static postural changes should be one of the goals of professionals working with child and adolescent health, as growth spurts occur at these age groups, and these are critical times for the onset of back problems^7^ caused by several adjustments, adaptations, and psychosocial and physical changes that are characteristic of this phase of development, in addition to intrinsic and extrinsic factors such as genetic, environmental, physical, emotional and socioeconomic factors.^8^

In this context, some studies have sought to identify the postural pattern of young individuals at school age, and their results suggest a high prevalence of anteroposterior and lateral changes in the spinal column,^9^^,^ 10 using photogrammetry to assess posture. Nevertheless, many of these studies are limited due to the lack of real knowledge on spinal column posture, which is only possible through radiological assessment. Thus, it is important to carry out studies that aim not only to assess static posture, but also to provide evidence of the actual positioning of the spinal column, in addition to knowledge of behavioral risk factors, such as postural habits. 

Thus, we assessed the hypothesis that inadequate postural habits in the seated position and when carrying the school backpack might be associated with the presence of static postural changes in the sagittal and frontal planes, respectively. Therefore, the aim of this study was to investigate if there is an association between behavioral risk factors, specifically postural habits, and the presence of structural postural changes in the spinal column of young individuals.

## Method

This is a cross-sectional study, and sample size was determined through data of mean and standard deviation of the spinal column lateral asymmetry angles (58.1±11.15), from the study of Bettany et al,^11^ thus requiring 58 individuals, with a confidence level of 95% and a sampling error of 5%. Therefore, 59 young individuals aged between 7 and 18 years, mean age of 12.9±2.3 years, 55.9% of which were females, were evaluated. These young individuals attended schools registered at the Family Health Strategy (FHS) program of Porto Alegre, and those referred to panoramic radiographic examination of the spinal column between the months of October and December 2012 were invited to participate in the study. Digital radiographs were performed at the Mãe de Deus Hospital in Porto Alegre, RS. 

The assessment method used was the Back Pain and Body Posture Evaluation Instrument (BackPEI) questionnaire, which has been validated with high reproducibility.^12^ The BackPEI questionnaire consists of 21 closed questions, which address the occurrence, frequency and intensity of pain in the last three months, as well as questions on demographic (age, gender) and behavioral factors (level of exercise, competitive or non-competitive practice of exercise, daily hours watching television and using the computer, number of daily hours of sleep, reading and/or studying in bed, and postures in activities of daily living); and panoramic X-ray examination of the spinal column in the right profile and anteroposterior view for evaluation of Cobb angles.^13^ Both evaluation procedures were performed on the same day and shift.

Based on the digital radiographs, Cobb angles were calculated using Matlab^(r)^ 7.9 software (MATrix LABoratory L.L.C, Dubai, UAE). For calculation of the thoracic kyphosis angle, the upper vertebral plateau of the first thoracic vertebra (T1) and the lower vertebral plateau of the 12^th^ thoracic vertebra (T12) were identified,^14^ and to calculate the lumbar lordosis angle the upper vertebral plateau of the first lumbar vertebra (L1) and the lower vertebral plateau of the fifth lumbar vertebra (L5) were used.^15^ The upper plateau of the cranial vertebra with the greatest tilt and the lower plateau of the caudal vertebra with the greatest tilt were used as a reference to calculate the angle of scoliosis.^16^ All calculations were performed by two independent examiners, and in cases where values between examiners differed by more than 5°, a third examiner performed a new assessment, with mean values between assessments being used for the analyses. The reproducibility of Cobb angles was tested by three independent examiners. The results showed excellent levels of intraobserver reproducibility for thoracic kyphosis (ICC=0.96, *p*<0.001); lumbar lordosis (ICC=0.98, *p*<0.001) and scoliosis (ICC=0.75, *p*<0.001). The interobserver reproducibility also showed excellent correlation for thoracic kyphosis (ICC=0.81, *p*<0.001) and lumbar lordosis (ICC=0.92, *p*<0.001), and moderate correlation for scoliosis (ICC=0.73, *p*<0.001). 

The proposed limits for children were used to classify sagittal spinal curvatures. The normal values for thoracic kyphosis were 20-50°.^14^ For lumbar lordosis, the 31-49.5° interval was adopted as normal.^17^ For statistical analysis, subjects were divided into two groups: (1) normal curvature and (2) postural change. In the sagittal plane, the postural change group comprised individuals who had both increases and decreases of curvatures. In the frontal plane, the postural change group consisted of individuals with scoliosis >10°.^10^

Data were analyzed using the Statistical Package for Social Sciences, version 18.0 (SPSS Inc. Released 2009. PASW Statistics for Windows, Version 18.0. Chicago, USA), based on the descriptive statistics and the chi-square test of association (bivariate analysis). Three analyses were performed, separately, for each dependent variable: (1) thoracic kyphosis, (2) lumbar lordosis, and (3) scoliosis, considering the demographic and behavioral variables as independent ones. The independent variables that had a level of significance of *p*<0.25 in the bivariate analysis were included in the Poisson regression model with robust variance separately for the outcomes thoracic kyphosis, lumbar lordosis and scoliosis. The measure of effect used was the Prevalence Ratio with their respective 95% confidence intervals (95% CI) (a=0.05). 

This study was approved by the Ethics Committee of Universidade Federal do Rio Grande do Sul, under number 19685, and students were included only after parents or guardians consented to their participation in the study by signing the free and informed consent form.

## Results

Table 1 shows the descriptive data of the sample stratified by age group. Considering that the age range of the sample has a broad spectrum, we investigated the prevalence of daily habits in the different age groups (Table 2). In this analysis, it can be observed that, in most cases, the evaluated habits had similar prevalence, regardless of age range. 

**Table 1:** Anthropometric characteristics and curvatures of the spinal column in mean and standard deviation, stratified by age group.

Age (years)	n	Weight (kg)	Height (cm)	BMI	Kyphosis (degrees)	Lordosis (degrees)	Scoliosis^a^ (degrees)
7 to 10	10	38.77±13.27	1.37±0.15	20.30±4.01	45.65±8.97	42.15±8.39	8.06±2.27
11 to 14	38	48.27±9.07	1.53±0.85	20.42±2.85	49.55±9.76	44.92±8.79	10.16±3.71
15 to 18	11	61.74±11.62	1.65±0.11	22.50±3.01	49.00±14.92	44.92±6.64	8.70±1.77
Total	59	48.55±12.17	1.52±0.13	20.71±3.15	48.74±10.45	44.41±8.37	9.58±3.32

^a^ Angle measured in 28 schoolchildren with scoliosis.

**Table 2:** Frequency of behavioral variables, stratified by age groups.

	7 to 10 yrs. (%)	11 to 14 yrs. (%)	15 to 18 yrs. (%)
Physical exercise practice			
Yes	80.0	89.5	90.9
No	20.0	10.5	9.1
Frequency of physical exercise			
1 to 2 days/week	57.1	53.6	44.4
3 or more days/week	42.9	46.4	55.6
Competitive physical exercise practice			
Yes	50.0	54.5	30.0
No	50.0	45.5	70.0
Time of TV/day			
0 to 3 hrs./day	50.0	57.6	72.7
4 to 7 hrs./day	25.0	27.3	0.
8 or more hrs./day	25.0	15.2	27.3
Time at computer/day			
0 to 3 hrs./day	83.3	71.4	55.6
4 or more hrs./day	16.7	28.6	44.4
Time of sleep per night			
0 to 7 hrs./day	20.0	34.4	62.5
8 to 9 hrs./day	40.0	40.6	25.0
10 or more hrs./day	40.0	25.0	12.5
Posture when sleeping			
Lateral decubitus	80.0	54.1	55.6
Dorsal decubitus	10.0	13.5	0
Ventral decubitus	10.0	32.4	44.4
Reads and/or studies in bed			
No	40.0	29.7	45.0
Yes	60.0	70.3	54.5
Posture in the seated position while writing			
Adequate	20.0	13.5	0.
Inadequate	80.0	86.5	100.0.
Posture in the seated position, on a stool			
Adequate	22.2	13.5	18.2
Inadequate	77.8	86.5	81.8
Posture in the seated position at the computer			
Adequate	14.3	16.2	0.
Inadequate	85.7	83.8	100.0.
Posture to pick object from the floor			
Adequate	20.0	13.5	10.0
Inadequate	80.0	86.5	90.0
Carrying school supplies			
Backpack with two straps	90.0	84.2	81.8
Others (briefcase, purse and others)	10.0	15.8	18.2
Carrying the school backpack			
Symmetrical straps on the shoulders	77.8	78.1	88.9
Non-symmetrical	22.2	21.9	11.1
Presence of back pain			
No	44.4	30.3	0.
Yes	55.6	69.7	100.0.

Considering these data, we chose to perform the analysis on the total sample. Thus, of the 59 subjects assessed, 30 had thoracic kyphosis, 19 had lumbar lordosis and 28 scoliosis. The association between demographic and behavioral variables with each postural change is shown separately for the sagittal (Table 3) and frontal (Table 4) planes.

**Table 3:** Results of association and prevalence ratios (PR) for the dependent variables thoracic kyphosis and lumbar lordosis, according to independent demographic and behavioral variables.

Variables	N (%)	Thoracic kyphosis				Lumbar lordosis		
		N (%)	*p* value	PR (95%CI)		N (%)	*p *value	PR (95%CI)
Gender (n=59)								
Male	33 (55.9)	13 (39.4)	0.041*	1		8 (24.2)	0.135	1
Female	26 (44.1)	17 (65.4)		1.18 (1.01-1.39)		11 (42.3)		1.14 (0.95-1.36)
Age groups (n=59)								
7 to 10 years	10 (16.9)	4 (40.0)	0.754	1		2 (20.0)	0.223	1
11 to 14 years	38 (64.4)	20 (52.6)		1.09 (0.85-1.38)		15 (39.5)		1.16 (0.92-1.47)
15 to 18 years	11 (18.6)	6 (54.5)		1.11 (0.82-1.47)		2 (18.2)		0.98 (0.74-1.31)
Behavioral								
Physical exercise practice (n=59)								
Yes	52 (88.1)	27 (51.9)	0.657	1		17 (32.9)	0.824	1
No	7 (11.9)	3 (42.9)		0.94 (0.71-1.23)		2 (28.6)		0.96 (0.74-1.27)
*Frequency of physical exercise (n=44)* ^c^								
1 to 2 days/week	23 (52.3)	15 (65.2)	0.028*	1		7 (30.4)	0.591	1
3 or more days/week	21 (47.7)	7 (33.3)		0.81 (0.66-0.97)		8 (38.1)		1.05 (0.86-1.31)
*Competitive physical exercise practice (n=51)* ^c^								
Yes	25 (49.0)	11 (44.0)	0.325	1		10 (40.0)	0.317	1
No	26 (51.0)	15 (57.7)		1.09 (0.91-1.31)		7 (26.9)		0.90 (0.74-1.09)
Time of TV/day (n=52)								
0 to 3 hrs./day	31 (59.6)	14 (45.2)	0.539	1		9 (29.0)	0.277	1
4 to 7 hrs./day	11 (21.2)	7 (63.4)		1.12 (0.91-1.39)		6 (54.5)		1.19 (0.95-1.51)
8 or more hrs./day	10 (19.2)	5 (50.0)		1.03 (0.81-1.33)		3 (30.0)		1.00 (0.78-1.29)
Time at computer/day (n=43)								
0 to 3 hrs./day	30 (69.8)	13 (43.3)	0.021*	1		11 (36.7)	0.911	1
4 or more hours/day	13 (30.2)	10 (76.9)		1.23 (1.03-1.47)		5 (38.5)		1.01 (0.81-1.27)
Time of sleep per night (n=50)								
0 to 7 hours/day	18 (36.0)	7 (38.9)	0.008	1		5 (27.8)	0.446	1
8 to 9 hours/day	19 (38.0)	6 (31.6)		0.94 (0.75-1.18)		5 (26.3)		0.98 (0.78-1.23)
10 or more hours/day	13 (26.0)	10 (76.9)		1.27 (1.03-1.56)		6 (46.2)		1.14 (0.89-1.46)
Posture when sleeping (n=56)								
Lateral decubitus	33 (58.9)	16 (48.5)	0.151	1		8 (24.2)	0.112	1
Dorsal decubitus	6 (10.7)	2 (33.3)		0.89 (0.66-1.21)		2 (33.3)		1.07 (0.79-1.45)
Ventral decubitus	17 (30.4)	16 (70.6)		1.14 (0.97-1.36)		9 (52.9)		1.23 (1.00-1.49)
Reads and/or studies in bed (n=58)								
No	20 (62.5)	9 (45.0)	0.582	1		4 (20.0)	0.109	1
Yes	12 (37.5)	20 (52.6)		1.05 (0.87-1.26)		15 (39.5)		1.16 (0.96-1.39)
Posture in the seated position while writing (n=58)								
Adequate	7 (12.1)	1 (14.3)	0.014*	1		2 (28.6)	0.879	1
Inadequate	51 (87.9)	28 (54.9)		1.35 (1.06-1.72)		16 (31.4)		1.02 (0.77-1.34)
Posture in the seated position on a stool (n=57)								
Adequate	9 (15.8)	1 (11.1)	0.001*	1		2 (22.2)	0.484	1
Inadequate	48 (84.2)	27 (56.2)		1.41 (1.14-1.72)		16 (33.3)		1.09 (0.85-1.39)
Posture in the seated position at the computer (n=55)								
Adequate	7 (12.7)	2 (28.6)	0.234	1		4 (57.1)	0.130	1
Inadequate	48 (87.3)	25 (52.1)		1.18 (0.89-1.55)		14 (29.2)		0.82 (0.63-1.05)
Posture to pick object from the floor (n=57)								
Adequate	8 (14.0)	4 (50.0)	0.957	1		2 (25.0)	0.654	1
Inadequate	49 (86.0)	24 (49.0)		0.99 (0.77-1.27)		16 (32.7)		1.06 (0.81-1.37)
*Carrying school supplies (n=59)* ^a^								
Backpack with two straps	50 (84.7)	23 (46.0)	0.032*	1		17 (34.0)	0.458	1
Others	9 (15.3)	7 (77.8)		1.21 (1.01-1.45)		2 (22.2)		0.91 (0.71-1.16)
*Carrying the school backpack (n=50)* ^c^								
Adequate	40 (80.0)	19 (47.5)	0.671	1		11 (27.5)	0.042	1
Inadequate	10 (20.0)	4 (40.0)		0.94 (0.74-1.20)		6 (60.0)		1.25 (1.01-1.56)
Presence of back pain (n=52)								
No	14 (26.9)	5 (35.7)	0.212	1		4 (28.6)	0.696	1
Yes	38 (73.1)	21 (55.3)		1.14 (0.92-1.41)		13 (34.2)		1.04 (0.84-1.29)

**Table 4:** Prevalence ratios (PR) for the dependent variable postural change in the frontal plane (scoliosis), according to independent demographic and behavioral variables.

	Total n (%)	With scoliosis n (%)	*p* value	PR (95%CI)
Demographic				
Gender (n=59)				
Male	33 (55.9)	12 (36.4)	0.058	1
Female	26 (44.1)	16 (61.5)		1.69 (0.98-2.91)
Age groups (n=59)				
7-10 yrs.	10 (16.9)	5 (50.0)	0.978	1
11-14 yrs.	38 (64.4)	18 (47.4)		0.94 (0.46-1.91)
15-18 yrs.	11 (18.6)	5 (45.5)		0.91 (0.37-2.22)
Behavioral				
Physical exercise practice (n=59)				
Yes	52 (88.1)	23 (44.2)	0.093	1
No	7 (11.9)	5 (71.4)		1.61 (0.92-2.82)
*Weekly frequency of physical exercise (n=44)* ^c^				
1-2 days/week	23 (52.3)	12 (52.2)	0.541	1
3 or more days/week	21 (47.7)	9 (42.9)		0.82 (0.43-1.54)
*Competitive physical exercise practice (n=51)* ^c^				
Yes	25 (49.0)	7 (28.0)	0.046	1
No	26 (51.0)	15 (57.7)		2.06 (1.01-4.18)
Time spent watching TV/day (n=52)				
0-3 hrs./day	31 (59.6)	14 (45.2)	0.852	1
4-7 hrs./day	11 (21.2)	6 (54.5)		1.20 (0.62-2.34)
8 or more hrs./day	10 (19.2)	5 (50.0)		1.10 (0.53-2.3)
Time at the computer/day (n=43)				
0-3 hours/day	30 (69.8)	15 (50.0)	0.507	1
4 or more hours/day	13 (30.2)	5 (38.5)		0.76 (0.35-1.67)
Time of sleep per night (n=50)				
0-7 hours a day	18 (36.0)	5 (27.8)	0.004	1
8-9 hours a day	19 (38.0)	9 (47.4)		1.71 (0.71-4.12)
10 or more hours a day	13 (26.0)	11 (84.6)		3.04 (1.39-6.64)
Posture when sleeping (n=56)				
Lateral decubitus	33 (58.9)	12 (36.4)		1
Dorsal decubitus	6 (10.7)	5 (83.3)	0.019	2.29 (1.28-4.07)
Ventral decubitus	17 (30.4)	10 (58.8)		1.61 (0.88-2.95)
Reads and/or studies in bed (n=58)				
No	20 (34.5)	7 (35.0)	0.232	1
Yes	38 (65.5)	20 (52.6)		1.50 (0.77-2.93)
Posture in the seated position while writing (n=58)				
Adequate	7 (12.1)	1 (14.3)	0.161	1
Inadequate	51 (87.9)	27 (52.9)		3.71 (0.59-23.1)
Posture seated on a stool (n=57)				
Adequate	9 (15.8)	4 (44.4)	0.768	1
Inadequate	48 (84.2)	24 (50.0)		1.12 (0.51-2.36)
Posture in the seated position at the computer (n=55)				
Adequate	7 (12.7)	3 (42.9)	0.737	1
Inadequate	48 (87.3)	24 (50.0)		1.16 (0.47-2.87)
Posture to pick object from the floor (n=57)				
Adequate	8 (14.0)	4 (50.0)	0.957	1
Inadequate	49 (86.0)	24 (49.0)		0.98 (0.46-2.07)
Carrying school supplies (n=59)				
Backpack with two straps	50 (84.7)	23 (46.0)	0.573	1
Others (briefcase, purse and others)	9 (15.3)	5 (55.6)		1.20 (0.62-2.33)
*Carrying the school backpack (n=50)* ^c^				
Adequate (symmetrical straps on the shoulders)	40 (80.0)	17 (42.5)	0.277	1
Inadequate (non-symmetrical)	10 (20.0)	6 (60.0)		1.41 (0.75-2.62)
Presence of back pain (n=52)				
No	14 (26.9)	5 (35.7)	0.477	1
Yes	38 (73.1)	18 (47.4)		1.32 (0.60-2.88)

**Figure 1:**
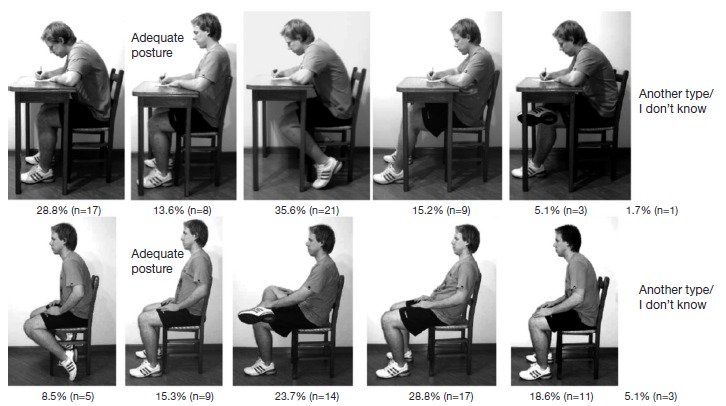
Percentages of postures adopted in the seated position, according to the BackPEI questionnaire by Noll et al12 (authorized image).

Considering the association found between inadequate postures in the seated position with thoracic kyphosis (Table 3), we sought to identify the inadequacies identified in young individuals while performing these postures. Figure 1 shows the percentages of young individuals for each of the inadequate postures in the seated position.

## Discussion

The main results show that thoracic kyphosis was associated with inadequate posture in the seated position when writing and sitting on a stool. Moreover, the habit of using the computer for 4 hours or more was also associated with these changes (Table 3), confirming the initial hypothesis of this study. 

In the study by Detsch et al,^9^ carried out only with girls, the habit of watching TV for 10 hours or more was associated with the presence of postural changes in the sagittal plane, although the same did not occur with the sitting posture at school. Nevertheless, these authors observed that most students had poor posture while sitting to study. Another similar study found no significant results for the association between the habit of watching television for more than two hours daily and postural changes.^2^ Although there is no consensus, it is believed that the time spent in an inadequate seated position can be considered a risk factor for the development of postural changes in the sagittal plane. Moreover, it is noteworthy that when specifically analyzing postural inadequacies in the seated position, whether when writing or sitting on a stool, it was observed that, for all inadequate postures, there is a tendency among young individuals to perform a trunk flexion (Fig. 1). Therefore, to establish preventive actions in schools, it is necessary to understand this characteristic, and thus the health professional should aim to minimize this bad postural habit. These findings do not allow us to state that there is a cause/effect association of postural change, and new longitudinal studies aimed at exploring this subject are necessary. 

Another association found with thoracic kyphosis was the female gender, with a prevalence ratio of 1.18.Another study also found increased incidence of kyphosis in girls.^9^ Vasconcelos et al^10^ found a high prevalence of dorsal hyperkyphosis in a sample of deaf children (75%) and suggested this change could be associated with natural physiological changes in growth and in the child's individual development. In this sense, it is considered that girls had higher propensity to develop thoracic hyperkyphosis because of a tendency to adopt a stooped posture to hide the development of breasts.^18^ Furthermore, the literature has shown that female gender can be considered a risk for the development of postural changes in the frontal plane. Vasconcelos et al^10^ reported an association between female gender and the presence of scoliosis, with an odds ratio of 3:1. Leal et al^19^ found a ratio of 1.28:1 and Nery et al,^20^ a ratio of 2.4:1, when comparing the female and male genders. However, in the present study, this association was not observed. Nevertheless, it is possible to observe a higher prevalence of scoliosis in females (61.5%) when compared to males (36.4%).

Among behavioral aspects, physical exercise showed no association with postural changes in the sagittal and frontal planes, although other studies have shown increased risk of postural changes with physical activity,^21^^,^ 22 and individuals who performed physical exercise three or more days a week were less likely to have thoracic kyphosis changes. The competitive practice of physical exercise showed association only with the presence of scoliosis**. **Meliscki et al,^21^ who evaluated swimmers aged 13-28 years, found a prevalence of scoliosis in females associated to the side where swimmers breathed and the type of swimming stroke they practiced, demonstrating the influence of sports practice on the positioning of anatomical structures, facilitating postural changes. Also, Santo et al,^22^ when analyzing school-age children, found an association between the presence of scoliosis and the practice of physical activity. It is noteworthy that, in the present study, we did not investigate the type of sport each subject practiced, thus limiting greater clarification on this issue. However, it is speculated that physical activity can be both a protective factor and a risk for postural changes. Possibly, factors such as the type of sport practiced, the volume of weekly training, time of practice and the way the activity is performed can influence the type of musculoskeletal response. 

As for the transport of school supplies, there was association between lumbar lordosis changes and how the backpack was carried, as well as thoracic kyphosis changes and how the school supplies were carried. These results differ from the initial study hypothesis that the inappropriate use of the backpack would be associated with the presence of scoliosis. The literature has also described the lack of association between postural changes and carrying school supplies;^9^^,^ 10 however, a tendency to postural change has been reported in students carrying school supplies inadequately.^9^ Lemos et al (2005)^23^ investigated the locations more often affected by postural changes when children carry loads of less, equal to and of more than 10% of body weight and found that postural changes begin to occur when the load is greater than 10% of body weight. The changes found in this study may be related to the weight of the school backpack, a variable that was not analyzed, which is a limitation of our study.

Another factor investigated was sleep duration, and it was observed that individuals who slept 10 or more hours a night showed an association with changes in thoracic kyphosis and scoliosis. Auvinen.^24^ found that insufficient sleep time (six hours or less) predisposed to low back pain. Paananen et al^25^ also reported that less than seven hours of sleep predisposed to postural changes. Furthermore, there have been reports that sleeping in the prone position generated higher incidence of postural changes in the sagittal plane, and that scoliosis was more prevalent in subjects who slept in the supine position, and yet there was no association in the study by Vasconcelos et al^10^ between postural changes and the position assumed when sleeping.^10^ In contrast, the present investigation identified an association between the habit of sleeping in the supine position and the chance of developing scoliosis, but it is believed that the existing data so far are inconsistent to define this association. Nevertheless, it seems that the adequate time of sleep (approximately 8 hours) may be considered a protective factor for the development of postural changes, which is in agreement with Auvinen et al,^24^ who recommends 8 or 9 hours of sleep/day. 

Finally, regarding the variable back pain, no association was found with postural change. This result is different from some studies in the literature, which found an association between thoracic kyphosis and back pain.^10^^,^ 26 Santo et al^22^ found a higher prevalence of back pain in female students and those whose parents had back pain. That is, literature shows conflicting results on the factors associated with back pain, such as hereditary and demographic aspects and postural change. These discrepancies may be related to local and regional characteristics of the study population. Thus, one demonstrates the importance of carrying out local studies to investigate the prevalence of changes and their risk factors, as it is not appropriate to generalize the results from different locations, since the variables pain and posture undergo significant cultural and social influence.

An important result obtained in this study refers to the high prevalence of postural changes in the assessed subjects, observed in 79.7% (n=47) of young individuals, with 47.5% (n=28) of them showing changes in the frontal plane, and 61% (n=36) in the sagittal plane.

Vasconcelos et al^10^ observed prevalence of 90.6% of postural changes in deaf children. Detsch et al,^9^ when assessing female students aged between 14 and 18 years, reported a prevalence of 66% for lateral changes and of 70% for anteroposterior changes in the municipality of São Leopoldo, RS. The same authors obtained similar results when assessing children aged 6 to 17 years from the municipality of Novo Hamburgo, RS, finding postural changes in 70.78% of the cases.^6^ High prevalence of postural changes was also found in a study that evaluated students from the first to fourth grades of elementary school in the municipality of Jaguariúna, São Paulo, which reported asymmetry or postural change in 98% of assessed individuals.^27^ These literature data corroborate the findings of this study, as all showed high prevalence of postural changes. Furthermore, the two studies that investigated changes in the plans separately indicated a greater frequency of changes in the sagittal plane. It is noteworthy that all the aforementioned studies assessed external postural changes, as they used noninvasive evaluation. This research used the gold standard to identify postural changes, and thus it is understood that the clinical relevance of this study derives from the knowledge of the actual position of the spine of young individuals, and it can be inferred that these showed high prevalence of structural spinal changes. 

However, the present study also showed limitations, such as the smaller sample size of the age ranges 7-10 years and 15-18 years, which hindered the separate statistical analysis by age groups. Also, the cross-sectional nature of the study does not allow us to analyze the cause/effect association of postural changes and habits. Additionally, the external and internal validity of the sample is limited regarding the prevalence of postural changes, as the sample was not randomly generated, but consisted of students referred for radiological examination. Nevertheless, the fact that the postural changes were evaluated with the gold standard compensates to some extent the lack of sample randomization and increases the internal study validity. Thus, the perspective is to carry out a cohort study in order to understand the factors that lead young individuals exposed to the school environment to develop postural changes.

Given the abovementioned facts, we found a high prevalence of postural structural changes in the spinal column and inadequate postural habits among the young individuals assessed, regardless of the age range. The results showed that of the 59 young individuals, 30 showed thoracic kyphosis change, which is associated with female gender, the practice of physical exercises only once or twice a week, more than 10 hours of sleep/day, inadequate posture in the seated position and how school supplies were carried; 19 showed abnormalities in the lumbar lordosis, which is associated with the act of carrying the school backpack asymmetrically, and 28 young individuals were diagnosed with scoliosis, which was associated with the practice of competitive sports and more than 10 hours of sleep/day. Thus, it is suggested that postural habits may be associated with postural changes, and the development of health policies to reduce the occurrence of bad postural habits is important.
