# Supplementary material for: Risk factors associated with structural postural changes in the spinal column of children and adolescents
Source: Rev Paul Pediatr. 2015 Mar;33(1):72–81. doi: 10.1016/j.rpped.2014.11.012 (PMC4436959; doi:10.1016/j.rpped.2014.11.012)
Supplement: Translation

# Translation: Fatores de risco associados a alterações posturais estruturais da coluna vertebral em crianças e adolescentes

**Authors:** Juliana Adami Sedrez, Maria Izabel Zaniratti da Rosa, Matias Noll, Fernanda da Silva Medeiros, Claudia Tarragô Candotti

**Affiliations:** Universidade Federal do Rio Grande do Sul (UFRGS), Porto Alegre, RS, Brasil

**Keywords:** Fatores de risco, Postura, Coluna vertebral/anormalidades, Criança, Adolescente, Epidemiologia

## Abstract

**OBJETIVO:**

Verificar se existe associação de fatores de risco comportamentais, especificamente hábitos posturais, com a presença de alteração postural estrutural na coluna vertebral de crianças e adolescentes.

**MÉTODOS:**

Foram avaliados 59 crianças e adolescentes, que responderam ao questionário auto-aplicável Back Pain and Body Posture Evaluation Instrument e fizeram o exame radiográfico panorâmico da coluna vertebral. De acordo com o ângulo de Cobb, as curvaturas sagitais da coluna vertebral foram classificadas como normais ou alteradas e, no plano frontal, como escolioses ou normais. Os dados foram analisados no SPSS 18.0, a partir de estatística descritiva e do teste de associação qui-quadrado (a=0,05).

**RESULTADOS:**

A prevalência de alterações posturais foi de 79,7% (n=47), 47,5% (n=28) apresentavam alteração no plano frontal e 61% (n=36) no sagital. Foi encontrada associação entre cifose torácica e sexo feminino, prática de exercício físico apenas uma ou duas vezes na semana, tempo de sono superior a 10 horas, posturas inadequadas para sentar no banco e sentar para escrever e o meio de transporte do material escolar. Para lordose lombar, observou-se associação com o transporte da mochila escolar de modo inadequado (assimétrico). Houve associação significativa entre a presença de escoliose com a prática de esporte competitivo e o tempo de sono superior a 10 horas.

**CONCLUSÕES:**

Hábitos de vida podem estar associados a alterações posturais, é importante o desenvolvimento políticas de saúde a fim de reduzir a prevalência de alterações posturais por meio da redução dos fatores de risco associados.

## Introdução

As alterações posturais estáticas são consideradas um problema de saúde pública, principalmente as que atingem a coluna vertebral, pois podem ser um fator predisponente às condições degenerativas da coluna vertebral do adulto.^1^^,^ 2 ^and^ 3 Além disso, a depender da sua magnitude, são capazes de gerar algum tipo de incapacidade para as atividades diárias.

As fases da infância e adolescência correspondem àquelas em que os jovens frequentam o ambiente escolar, no qual permanecem longos períodos sentados, normalmente em uma postura inadequada e, na maioria das vezes, em mobiliários inadequados^4^ que, somados à tendência de um estilo de vida sedentário adotado na fase escolar,^5^ podem também favorecer o surgimento das alterações posturais estáticas. Além disso, parece existir uma tendência de que os hábitos posturais adotados durante a infância e adolescência poderão se refletir na vida adulta dos jovens.^6^

Dessa forma, investigações sobre a ocorrência de alterações posturais estáticas e das variáveis associadas a essa condição ajudam a compreender os fatores de risco para os problemas de coluna. A detecção precoce dessas alterações é o primeiro passo para a prevenção das condições predisponentes ao aparecimento desses problemas. Assim, detectar precocemente as alterações posturais estáticas deveria ser um dos objetivos dos profissionais que atuam na saúde da criança e do adolescente, visto que, nessas faixas etárias, ocorrem os estirões de crescimento, momentos críticos para o aparecimento dos problemas de coluna,^7^ decorrentes dos vários ajustes, adaptações e mudanças corporais e psicossociais característicos dessa fase do desenvolvimento, além de fatores intrínsecos e extrínsecos, como hereditariedade, ambiente, condições físicas, fatores emocionais e socioeconômicos.^8^

Nesse contexto, alguns estudos têm buscado identificar o padrão postural de jovens em idade escolar e seus resultados sugerem alta prevalência de alterações ântero-posteriores e laterais na coluna vertebral^9^ ^and^ 10 com o uso da fotogrametria como meio de avaliação da postura. Não obstante, muitos desses estudos apresentam como limitação o não conhecimento real da postura da coluna vertebral, somente possível por meio da radiologia. Dessa forma, é importante conduzir estudos que visem não somente avaliar a postura estática, mas também fornecer evidências do real posicionamento da coluna vertebral, além do conhecimento dos fatores de risco comportamentais, tais como os hábitos posturais. Elaborou-se, então, a hipótese de que os hábitos inadequados na postura sentada e ao carregar a mochila escolar estariam associados à presença de alteração postural estática no plano sagital e frontal, respectivamente. Portanto, o objetivo do presente estudo foi verificar se existe associação de fatores de risco comportamentais, especificamente os hábitos posturais, com a presença de alteração postural estrutural na coluna vertebral de jovens.

## Método

Estudo transversal. O tamanho amostral foi determinado por meio de dados de média e desvio padrão dos ângulos de assimetria lateral da coluna vertebral (58,1±11,15) do estudo de Bettany et al.,^11^ foram necessários 58 indivíduos, foi adotado nível de confiança de 95% e erro amostral de 5%. Desse modo, foram avaliados 59 jovens entre sete e 18 anos, idade média de 12,9±2,3 anos, 55,9% do sexo masculino. Tais jovens frequentavam escolas cadastradas em uma Estratégia Saúde da Família (ESF) de Porto Alegre e aqueles que tinham encaminhamento médico para exame radiológico panorâmico da coluna vertebral entre outubro a dezembro de 2012 foram convidados a participar do estudo. As radiografias digitais foram feitas no Hospital Mãe de Deus, em Porto Alegre (RS).

Os métodos de avaliação usados foram: questionário Back Pain and Body Posture Evaluation Instrument (BackPEI), validado e com altos índices de reprodutibilidade.^12^ O BackPEI é constituído por 21 questões fechadas, que abordam a ocorrência, a frequência e a intensidade da dor nas costas nos últimos três meses, bem como perguntas demográficas (idade, sexo), comportamentais (nível de exercício físico, prática competitiva ou não de exercício físico, tempo diário assistindo à televisão e usando o computador, número de horas diárias de sono, ler e/ou estudar na cama e posturas nas atividades de vida diárias); e exame de raios-X panorâmico da coluna vertebral nas incidências perfil direito e posteroanterior para avaliação dos ângulos de Cobb.^13^ Ambos os procedimentos de avaliação foram feitos no mesmo dia e turno.

A partir das radiografias digitais, foram calculados os ângulos de Cobb no *software* Matlab^(r)^ 7.9. Para os cálculos do ângulo da cifose torácica, foram marcados o platô vertebral superior da primeira vértebra torácica (T1) e o platô vertebral inferior da 12ª vértebra torácica (T12) 14 e para o cálculo do ângulo da lordose lombar, foram usados o platô vertebral superior da primeira vértebra lombar (L1) e o platô vertebral inferior da quinta vértebra lombar (L5). 15 Para o cálculo do ângulo de escoliose foi usado como referência o platô superior da vértebra cranial mais inclinada e o platô inferior da vértebra caudal mais inclinada. 16 Todos os cálculos foram feitos por dois avaliadores independentes e nos casos em que as medidas entre os avaliadores diferiram mais do que 5°, um terceiro avaliador fez uma nova avaliação. Foram usados para a análise os valores médios entre as avaliações. A reprodutibilidade dos ângulos Cobb foi testada por três avaliadores independentes. Os resultados demonstraram excelentes níveis de reprodutibilidade intra-avaliador para cifose torácica (ICC=0,96; *p*<0,001); lordose lombar (ICC=0,98; *p*<0,001) e escoliose (ICC=0,75; *p*<0,001). A reprodutibilidade interavaliador também apresentou correlação excelente para cifose torácica (ICC=0,81; *p*<0,001) e lordose lombar (ICC=0,92; *p*<0,001), e correlação moderada para escoliose (ICC=0,73; *p*<0,001).

Para classificar as curvaturas sagitais da coluna vertebral foram usados os limites propostos para crianças. Os valores de normalidade para cifose torácica foram 20-50°.^14^ Para a lordose lombar, adotou-se como normalidade o intervalo de 31-49,5°.^17^ Para análise estatística, os indivíduos foram reunidos em dois grupos: (1) curvatura normal e (2) alteração postural. No plano sagital, o grupo alteração postural foi composto por indivíduos que apresentavam tanto aumento quanto diminuição das curvaturas. No plano frontal, o grupo alteração postural foi composto pelos indivíduos com escolioses superiores a 10°.^10^

Os dados foram analisados no Statistical Package for the Social Sciences (SPSS) versão 18.0, a partir de estatística descritiva e do teste de associação qui-quadrado (análise bivariada). Foram feitas três análises, separadamente, uma para cada variável dependente: (1) cifose torácica, (2) lordose lombar e (3) escoliose e consideradas as variáveis demográficas e comportamentais como independentes. As variáveis independentes que apresentaram significância de p < 0,25 na análise bivariada foram incluídas no modelo de regressão de Poisson com variância robusta separadamente para os desfechos cifose torácica, lordose lombar e escoliose. A medida de efeito usada foi a razão de prevalência, com seus respectivos intervalos de confiança de 95% (IC95%) (a=0,05).

Este estudo foi aprovado pelo Comitê de Ética em Pesquisa da Universidade Federal do Rio Grande do Sul, sob o número 19.685, e os escolares só foram incluídos após os pais ou responsáveis consentirem com sua participação, por meio da assinatura do Termo de Consentimento Livre e Esclarecido.

## Resultados

A tabela 1 expressa os dados descritivos da amostra estratificados por faixa etária. Considerando que a faixa etária da amostra apresenta um espectro amplo, foi verificada a prevalência dos hábitos diários nos diferentes grupos etários (tabela 2). Nesta análise**,** observa-se que, em sua maioria, os hábitos avaliados apresentaram prevalência semelhante, independentemente da faixa etária.

**Tabela 1:** Características antropométricas e das curvaturas da coluna vertebral em média e desvio padrão, estratificadas por faixa etária

Idade (anos)	N	Peso (kg)	Estatura (cm)	IMC	Cifose (graus)	Lordose (graus)	Escoliose^a^ (graus)
7-10	10	38,77±13,27	1,37±0,15	20,30±4,01	45,65±8,97	42,15±8,39	8,06±2,27
11-14	38	48,27±9,07	1,53±0,85	20,42±2,85	49,55±9,76	44,92±8,79	10,16±3,71
15-18	11	61,74±11,62	1,65±0,11	22,50±3,01	49,00±14,92	44,92±6,64	8,70±1,77
Total	59	48,55±12,17	1,52±0,13	20,71±3,15	48,74±10,45	44,41±8,37	9,58±3,32

a Ângulo avaliado em 28 escolares com presença de escoliose.

**Tabela 2:** Frequência das variáveis comportamentais, estratificada por faixas etárias

	7 a 10 anos (%)	11 a 14 anos (%)	15 a 18 anos (%)
*Prática de exercício físico*			
Sim	80,0	89,5	90,9
Não	20,0	10,5	9,1

*Frequência de exercício físico*			
L-2 dias/semana	57,1	53,6	44,4
≥3 dias/semana	42,9	46,4	55,6

*Prática competitiva de exercício*			
Sim	50,0	54,5	30,0
Não	50,0	45,5	70,0

*Tempo de televisão por dia*			
0-3h/dia	50,0	57,6	72,7
4-7 h/dia	25,0	27,3	0
≥8h/dia	25,0	15,2	27,3

*Tempo de computador por dia*			
0-3h/dia	83,3	71,4	55,6
≥4h/dia	16,7	28,6	44,4

*Tempo de sono por noite*			
0-7h/dia	20,0	34,4	62,5
8-9h/dia	40,0	40,6	25,0
≥10h/dia	40,0	25,0	12,5

Decúbito *ao dormir*			
Lateral	80,0	54,1	55,6
Dorsal	10,0	13,5	0
Ventral	10,0	32,4	44,4

*Ler e/ou estudar na cama*			
Não	40,0	29,7	45,0
Sim	60,0	70,3	54,5

*Postura sentada para escrever*			
Adequada	20,0	13,5	0
Inadequada	80,0	86,5	100,0

*Postura sentada em um banco*			
Adequada	22,2	13,5	18,2
Inadequada	77,8	86,5	81,8

*Postura sentada no computador*			
Adequada	14,3	16,2	0
Inadequada	85,7	83,8	100,0

*Postura para pegar objeto do chão*			
Adequada	20,0	13,5	10,0
Inadequada	80,0	86,5	90,0

*Transporte do material escolar*			
Mochila	90,0	84,2	81,8
Outro	10,0	15,8	18,2

*Transporte da mochila escolar*			
Alças simétricas sobre os ombros	77,8	78,1	88,9
Modo não simétrico	22,2	21,9	11,1

*Dor nas costas*			
Não	44,4	30,3	0
Sim	55,6	69,7	100,0

Considerando esses dados, optou-se por fazer a análise em conjunto da amostra total. Assim, dos 59 indivíduos avaliados, 30 apresentaram cifose torácica, 19 lordose lombar e 28 escoliose. A associação entre variáveis demográficas e comportamentais com cada alteração postural é apresentada separadamente para os planos sagital (tabela 3) e frontal (tabela 4).

**Tabela 3:** Resultados de associação e razões de prevalência (RP) para as variáveis dependentes cifose torácica e lordose lombar, segundo as variáveis independentes demográficas e comportamentais

Variáveis	n (%)	Cifose torácica			Lordose lombar		
		n (%)	*p* valor	RP (IC95%)	n (%)	*p* valor	RP (IC95%)
**Demográficas**							
*Sexo (n=59)*							
Masculino	33 (55,9)	13 (39,4)	0,041	1	8 (24,2)	0,135	1
Feminino	26 (44,1)	17 (65,4)		1,18 (1,01-1,39)	11 (42,3)		1,14 (0,95-1,36)

*Grupos etários (n=59)*							
7-10 anos	10 (16,9)	4 (40,0)	0,754	1	2 (20,0)	0,223	1
11-14 anos	38 (64,4)	20 (52,6)		1,09 (0,85-1,38)	15 (39,5)		1,16 (0,92-1,47)
15-18 anos	11 (18,6)	6 (54,5)		1,11 (0,82-1,47)	2 (18,2)		0,98 (0,74-1,31)

**Comportamentais**							
*Prática de exercício físico (n=59)*							
Sim	52 (88,1)	27 (51,9)	0,657	1	17 (32,9)	0,824	1
Não	7 (11,9)	3 (42,9)		0,94 (0,71-1,23)	2 (28,6)		0,96 (0,74-1,27)

*Frequência de exercício físico (n=44)*							
L-2 dias/semana	23 (52,3)	15 (65,2)	0,028	1	7 (30,4)	0,591	1
≥3 dias/semana	21 (47,7)	7 (33,3)		0,81 (0,66-0,97)	8 (38,1)		1,05 (0,86-1,31)

*Prática competitiva de exercício (n=51)*							
Sim	25 (49.0)	11 (44.0)	0,325	1	10 (40.0)	0,317	1
Não	26 (51.0)	15 (57,7)		1,09 (0,91-1,31)	7 (26,9)		0.90 (0,74-1,09)

*Tempo de televisão por dia (n=52)*							
0-3h/dia	31 (59,6)	14 (45,2)	0,539	1	9 (29,0)	0,277	1
4-7h/dia	11 (21,2)	7 (63,4)		1,12 (0,91-1,39)	6 (54,5)		1,19 (0,95-1,51)
≥8h/dia	10 (19,2)	5 (50,0)		1,03 (0,81-1,33)	3 (30,0)		1,00 (0,78-1,29)

*Tempo de computador (n=43)*							
0-3h/dia	30 (69,8)	13 (43,3)	0,021	1	11 (36,7)	0,911	1
4h/dia	13 (30,2)	10 (76,9)		1,23 (1,03-1,47)	5 (38,5)		1,01 (0,81-1,27)

*Tempo de sono por noite (n=50)*							
07h	18 (36)	7 (38,9)	0,008	1	5 (27,8)	0,446	1
8-9h	19 (38)	6 (31,6)		0,94 (0,75-1,18)	5 (26,3)		0,98 (0,78-1,23)
≥10h	13 (26)	10 (76,9)		1,27 (1,03-1,56)	6 (46,2)		1,14 (0,89-1,46)

Decúbito *ao dormir (n=56)*							
Lateral	33 (58,9)	16 (48,5)		1	8 (24,2)		1
Dorsal	6 (10,7)	2 (33,3)	0,151	0,89 (0,66-1,21)	2 (33,3)	0,112	1,07 (0,79-1,45)
Ventral	17 (30,4)	16 (70,6)		1,14 (0,97-1,36)	9 (52,9)		1,23 (1,00-1,49)

*Ler e/ou estudar na cama (n=58)*							
Não	20 (62,5)	9 (45,0)	0,582	1	4 (20)	0,109	1
Sim	12 (37,5)	20 (52,6)		1,05 (0,87-1,26)	15 (39,5)		1,16 (0,96-1,39)

*Postura sentada para escrever (n=58)*							
Adequada	7 (12,1)	1 (14,3)	0,014	1	2 (28,6)	0,879	1
Inadequada	51 (87,9)	28 (54,9)		1,35 (1,06-1,72)	16 (31,4)		1,02 (0,77-1,34)

*Postura sentada em um banco (n=57)*							
Adequada	9 (15,8)	1 (11,1)	0,001	1	2 (22,2)	0,484	1
Inadequada	48 (84,2)	27 (56,2)		1,41 (1,14-1,72)	16 (33,3)		1,09 (0,85-1,39)

*Postura sentada no computador (n=55)*							
Adequada	7 (12,7)	2 (28,6)	0,234	1	4 (57,1)	0,130	1
Inadequada	48 (87,3)	25 (52,1)		1,18 (0,89-1,55)	14 (29,2)		0,82 (0,63-1,05)

*Postura para pegar objeto do chão (n=57)*							
Adequada	8 (14,0)	4 (50,0)	0,957	1	2 (25,0)	0,654	1
Inadequada	49 (86,0)	24 (49,0)		0,99 (0,77-1,27)	16 (32,7)		1,06 (0,81-1,37)

*Meio de transporte do material escolar (n=59)*							
Mochila	50 (84,7)	23 (46,0)	0,032	1	17 (34,0)	0,458	1
Outro	9 (15,3)	7 (77,8)		1,21 (1,01-1,45)	2 (22,2)		0,91 (0,71-1,16)

*Transporte da mochila escolar (n=50)*							
Adequado	40 (80,0)	19 (47,5)	0,671	1	11 (27,5)	0,042	1
Inadequado	10 (20,0)	4 (40,0)		0,94 (0,74-1,20)	6 (60,0)		1,25 (1,01-1,56)

*Dor nas costas (n=52)*							
Não	14 (26,9)	5 (35,7)	0,212	1	4 (28,6)	0,696	1
Sim	38 (73,1)	21 (55,3)		1,14 (0,92-1,41)	13 (34,2)		1,04 (0,84-1,29)

**Tabela 4:** Razões de prevalência (RP) para a variável dependente alteração postural no plano frontal (escoliose), segundo as variáveis independentes demográficas e comportamentais

	Total n (%)	Com escoliose n (%)	p valor	RP (IC95%)
**Demográficas**				
*Sexo (n=59)*				
Masculino	33 (55,9)	12 (36,4)	0,058	1
Feminino	26 (44,1)	16 (61,5)		1,69 (0,98-2,91)

*Grupos etários (n=59)*				
7-10 anos	10 (16,9)	5 (50,0)	0,978	1
11-14 anos	38 (64,4)	18 (47,4)		0,94 (0,46-1,91)
15-18 anos	11 (18,6)	5 (45,5)		0,91 (0,37-2,22)

**Comportamentais**				
*Prática de exercício físico (n=59)*				
Sim	52 (88,1)	23 (44,2)	0,093	1
Não	7 (11,9)	5 (71,4)		1,61 (0,92-2,82)

*Frequência de exercício físico (n=44)*				
1-2 dias/semana	23 (52,3)	12 (52,2)	0,541	1
3 ou mais dias/semana	21 (47,7)	9 (42,9)		0,82 (0,43-1,54)

*Prática competitiva de exercício físico (n=51)*				
Sim	25 (49)	7 (28)	0,046*	1
Não	26 (51)	15 (57,7)		2,06 (1,01-4,18)

*Tempo assistindo televisão por dia (n=52)*				
0-3h/dia	31 (59,6)	14 (45,2)	0,852	1
4-7h/dia	11 (21,2)	6 (54,5)		1,20 (0,62-2,34)
≥8h/dia	10 (19,2)	5 (50)		1,10 (0,53-2,3)

*Tempo de uso do computador por dia (n=43)*				
0-3h/dia	30 (69,8)	15 (50)	0,507	1
≥4h/dia	13 (30,2)	5 (38,5)		0,76 (0,35-1,67)

*Tempo de sono por noite (n=50)*				
0-7h/dia	18 (36,0)	5 (27,8)	0,004*	1
8-9h/dia	19 (38,0)	9 (47,4)		1,71 (0,71-4,12)
≥10h/dia	13 (26,0)	11 (84,6)		3,04 (1.39-6,64)

Decúbito *ao dormir (n=56)*				
Lateral	33(58,9)	12 (36,4)		1
Dorsal	6(10,7)	5(83,3)	0,019*	2,29(1,28-4,07)
Ventral	17(30,4)	10(58,8)		1,61(0,88-2,95)

*Ler e/ou estudar na cama (n=58)*				
Não	20 (34,5)	7 (35,0)	0,232	1
Sim	38 (65,5)	20 (52,6)		1,50 (0,77-2,93)

*Postura sentada para escrever (n=58)*				
Adequada	7 (12,1)	1 (14,3)	0,161	1
Inadequada	51 (87,9)	27 (52,9)		3,71 (0,59-23,1)

*Postura sentada em um banco (n=57)*				
Adequada	9 (15,8)	4 (44,4)	0,768	1
Inadequada	48 (84,2)	24 (50,0)		1,12 (0,51-2,36)

*Postura sentada ao computador (n=55)*				
Adequada	7 (12,7)	3 (42,9)	0,737	1
Inadequada	48 (87,3)	24 (50,0)		1,16 (0,47-2,87)

*Postura para pegar objeto do chão (n=57)*				
Adequada	8 (14,0)	4 (50,0)	0,957	1
Inadequada	49 (86,0)	24 (49,0)		0,98 (0,46-2,07)

*Meio de transporte do material escolar (n=59)*				
Mochila de duas alças	50 (84,7)	23 (46,0)	0,573	1
Outro (pasta, bolsa e outros)	9 (15,3)	5 (55,6)		1,20 (0,62-2,33)

*Transporte da mochila escolar (n=50)*				
Adequado	40 (80,0)	17 (42,5)	0,277	1
Inadequado	10 (20,0)	6 (60,0)		1,41 (0,75-2,62)

*Dor nas costas (n=52)*				
Não	14 (26,9)	5 (35,7)	0,477	1
Sim	38 (73,1)	18 (47,4)		1,32 (0,60-2,88)

Considerando a associação encontrada entre as posturas inadequadas ao sentar com a cifose torácica (tabela 3), procurou-se conhecer as inadequações apresentadas pelos jovens durante a execução dessas posturas. A figura 1 mostra as porcentagens de jovens para cada uma das posturas inadequadas ao sentar.

**Figura 1:** Porcentagens das posturas adotadas ao sentar, segundo o questionário BackPEI de Noll et al.12 (Imagem autorizada).

## Discussão

Os principais resultados demonstram que a alteração de cifose torácica esteve associada à postura inadequada ao sentar para escrever e sentar em um banco. Além disso, o hábito de usar o computador por quatro horas ou mais também se associou a essas alterações (tabela 3), o que confirma a hipótese inicial desse estudo.

No estudo de Detsch et al.,^9^ feito apenas com meninas, o hábito de assistir a televisão por 10 horas ou mais se associou à presença de alterações posturais no plano sagital, embora o mesmo não tenha ocorrido com a postura sentada no colégio. Não obstante, esses autores perceberam que as alunas, em sua maioria, apresentavam uma postura inadequada ao sentar para estudar. Outro estudo semelhante não encontrou resultados significativos para associação entre o hábito de assistir televisão por mais de duas horas por dia e alteração postural.^2^ Apesar de não haver um consenso, acredita-se que o tempo de permanência na postura sentada de forma inadequada possa ser considerado um fator de risco para o desenvolvimento de alterações posturais no plano sagital. Além disso, cabe ressaltar que, ao analisar especificamente as inadequações posturais assumidas durante o hábito de sentar, tanto para escrever como em um banco, observou-se que para todas as posturas inadequadas há uma tendência dos jovens de assumir um padrão flexor de tronco (fig. 1). Dessa forma, para atuações preventivas no âmbito escolar, é necessário compreender essa característica, de forma que a atuação do profissional da saúde vise a minimizar esse mau hábito postural. Tais achados não permitem afirmar que exista uma relação causa/efeito da alteração postural. Seria interessante a feitura de novos estudos longitudinais que visem a explorar essa questão.

Outra associação encontrada com a alteração de cifose torácica foi ser do sexo feminino, com uma razão de prevalência de 1,18. Outro estudo também encontrou cifose aumentada em meninas.^9^ Vasconcelos et al.,^10^ ao encontrar elevada prevalência de hipercifose dorsal em amostra de crianças surdas (75%), sugeriram que essa alteração poderia estar associada às alterações fisiológicas naturais do crescimento e do desenvolvimento individual da criança. Nesse sentido, considera-se que o fato de meninas apresentarem maior propensão a desenvolver hipercifose torácica pode ser explicado pela tendência à adoção de postura curvada para esconder o desenvolvimento dos seios.^18^ Além disso, a literatura tem evidenciado que o sexo feminino pode ser considerado de risco para o desenvolvimento de alterações posturais no plano frontal. Vasconcelos et al.^10^ relataram associação entre o sexo feminino e a presença de escoliose, com razão de chances de 3:1. Leal et al.^19^ encontraram uma proporção de 1,28:1 e Nery et al.^20^ uma proporção de 2,4:1 quando comparados os sexos feminino e masculino. No entanto, no presente estudo não foi encontrada essa associação. Apesar disso, é possível observar uma maior prevalência de escoliose no sexo feminino (61,5%) em comparação com o masculino (36,4%).

Dentre os aspectos comportamentais, a prática de exercício físico não demonstrou associação com alterações posturais nos planos sagital e frontal, embora outros estudos tenham demonstrado maior risco de alterações posturais com a prática de atividade física^21^ ^and^ 22 e os indivíduos que praticavam exercício físico com frequência de três ou mais dias por semana tivessem menor chance de apresentar alterações de cifose torácica. A prática competitiva de exercício físico mostrou associação apenas com a presença de escoliose. Meliscki et al.,^21^ que avaliaram nadadores de 13 a 28 anos, encontraram uma prevalência de escoliose no sexo feminino associada ao lado em que as nadadoras respiravam e ao tipo de nado que praticavam, o que demonstra a influência da prática esportiva sobre o posicionamento das estruturas anatômicas e facilita alterações posturais. Ainda, Santo et al.,^22^ ao analisar crianças em idade escolar, encontraram associação entre a presença de escoliose e a prática de atividade física. Cabe ressaltar que, no presente estudo, não foi investigada a modalidade esportiva que cada sujeito praticava, o que limitou possíveis maiores aprofundamentos sobre essa questão. No entanto, especula-se que a prática de atividade física pode ser tanto um fator de proteção quanto de risco para as alterações posturais. Possivelmente, fatores como o tipo de modalidade praticada, o volume de treino semanal, o tempo de prática da modalidade e a maneira como a atividade é conduzida influenciem no tipo de resposta musculoesquelética.

Quanto ao transporte do material escolar, houve associação entre alterações na lordose lombar e o modo de transportar a mochila, bem como alterações na cifose torácica e no meio de transportar o material. Esses resultados diferem da hipótese inicial do estudo de que o uso inadequado da mochila estaria associado à presença de escoliose. A literatura também tem descrito ausência de associação entre alteração postural e o transporte de material escolar.^9^ ^and^ 10 No entanto, tem sido relatada uma tendência de alteração postural nas alunas que carregam o material escolar de forma inadequada.^9^ Lemos et al. (2005)^23^ investigaram as regiões mais frequentes das alterações posturais quando as crianças carregavam cargas menores, iguais e superiores a 10% do peso corporal, e encontraram que as mudanças posturais começam a ocorrer quando a carga é superior a 10% do peso corporal. As alterações encontradas nesse estudo podem estar associadas ao peso na mochila escolar, variável que não foi analisada, o que caracteriza uma limitação de nosso estudo.

Outro fator investigado foi o hábito ao dormir. Foi observado que os indivíduos que dormem 10 ou mais horas por noite apresentaram associação com alterações de cifose torácica e escoliose. Auvinen et al.^24^ encontraram que tempo insuficiente de sono (seis horas ou menos) predispõe à dor lombar. Paananen et al.^25^ também referem que tempo inferior a sete horas de sono predispõe a alterações posturais. Além disso, há descrição de que o decúbito ventral foi a posição adotada ao dormir que gerou maior incidência de alterações posturais no plano sagital e que a escoliose foi mais prevalente nos sujeitos que adotavam a postura de decúbito dorsal. Apesar disso, não houve, no estudo de Vasconcelos et al.,^10^ associação entre as alterações posturais e a posição usada para dormir.^10^ Ao contrário, na presente investigação identificou-se associação entre o hábito de dormir em decúbito dorsal e a chance de escoliose, mas acredita-se que os dados existentes até o momento são inconsistentes para definir tal associação. Não obstante, parece que o tempo de sono adequado (aproximadamente oito horas) pode ser considerado um fator de proteção para o desenvolvimento das alterações posturais, o que vai ao encontro do que recomendam Auvinen et al.,^24^ oito ou nove horas de sono por dia.

Por fim, quanto à variável dor nas costas, não foi encontrada associação com a alteração postural. Esse resultado é divergente de alguns estudos encontrados na literatura, os quais encontraram associação entre hipercifose torácica e dor nas costas.^10^ ^and^ 26 Já Santo et al.^22^ encontraram maior prevalência de dor nas costas nos escolares do sexo feminino e que tinham pais com dor nas costas. Ou seja, a literatura apresenta resultados divergentes sobre os fatores associados à dor nas costas, como aspectos hereditários, demográficos e alteração postural. Essas divergências podem estar relacionadas às características locais e regionais da população investigada. Dessa forma, demonstra-se a importância de fazer estudos locais para investigar a prevalência das alterações e seus fatores de risco, visto que não é adequado generalizar os resultados de diferentes localidades, pois as variáveis dor e postura sofrem importante influência cultural e social.

Importante resultado obtido no presente estudo refere-se à elevada prevalência de alterações posturais nos sujeitos avaliados, verificada em 79,7% (n = 47) dos jovens; 47,5% (n = 28) dos avaliados apresentavam alteração no plano frontal e 61% (n = 36) no sagital. Vasconcelos et al.^10^ obtiveram prevalência de 90,6% de alterações posturais em crianças surdas. Detsch et al.,^9^ ao avaliar escolares do sexo feminino, com idade entre 14 e 18 anos, relataram uma prevalência de 66% para as alterações laterais e de 70% para as anteroposteriores da cidade de São Leopoldo (RS). Os mesmos autores obtiveram resultados semelhantes ao avaliar escolares de seis a 17 anos da cidade de Novo Hamburgo (RS) e encontraram alteração postural em 70,78% dos casos.^6^ Elevada prevalência de alterações posturais também foi encontrada em um estudo que avaliou estudantes da 1ª à 4ª série do ensino fundamental de uma escola da cidade de Jaguariúna (SP), o qual referiu assimetria ou alteração postural em 98% dos avaliados.^27^ Esses dados de literatura corroboram os achados do presente estudo, visto que todos obtiveram índices elevados de prevalência de alterações posturais. Além disso, os dois estudos que investigaram alterações nos planos separadamente apontaram para a existência de alterações no plano sagital com maior frequência. Cabe ressaltar também que todos os estudos citados avaliaram as alterações posturais externas, já que usaram avaliação não invasiva. A presente pesquisa usou o padrão-ouro para identificar as alterações posturais e, com isso, entende-se que a relevância clínica deste estudo decorre do conhecimento do posicionamento real da coluna vertebral de jovens. Pode-se inferir que esses apresentaram elevada prevalência de alterações estruturais da coluna vertebral.

No entanto, o presente nosso estudo também apresentou limitações. Por exemplo, o menor tamanho amostral dos grupos etários de sete a 10 anos e de 15 a 18 anos, o que dificultou a análise estatística separadamente por grupos etários. Ainda, o caráter transversal do estudo não possibilita analisar a relação causa/efeito das alterações e dos hábitos posturais. Além disso, a validade externa e interna da amostra é limitada em relação à prevalência das alterações posturais, pois não foi composta de forma aleatória, e sim por estudantes encaminhados para fazer exame radiológico. Apesar disso, o fato de as alterações posturais terem sido avaliadas com o padrão-ouro compensa em algum grau a não aleatoridade da amostra e eleva a validade interna do estudo. Dessa forma, tem-se como perspectivas a feitura de um estudo de coorte, a fim de compreender quais os fatores que levam os jovens expostos ao ambiente escolar a desenvolver alterações posturais.

Diante do exposto, constatou-se elevada prevalência de alterações posturais estruturais da coluna vertebral e de hábitos posturais inadequados nos jovens estudados, independentemente da faixa etária estudada. Os resultados demonstraram que, dos 59 jovens, 30 apresentaram alteração na cifose torácica, associada ao sexo feminino, à prática de exercícios físico apenas uma ou duas vezes na semana, ao tempo de sono superior a 10 horas, à postura inadequada ao sentar e ao meio de transporte do material escolar; 19 apresentaram alteração na lordose lombar, associada ao ato de carregar a mochila de forma assimétrica; e em 28 jovens foi diagnosticada a escoliose, que se associou à prática de esporte competitivo e ao tempo de sono superior a 10 horas. Dessa forma, sugere-se que hábitos posturais possam estar associados com alterações posturais. É importante o desenvolvimento de políticas de saúde a fim de reduzir a ocorrência dos maus hábitos posturais.
